# Standardized radiographic interpretation of thoracic tuberculosis in children

**DOI:** 10.1007/s00247-017-3868-z

**Published:** 2017-08-29

**Authors:** Nathan David P. Concepcion, Bernard F. Laya, Savvas Andronikou, Pedro A. N. Daltro, Marion O. Sanchez, Jacqueline Austine U. Uy, Timothy Reynold U. Lim

**Affiliations:** 1 Section of Pediatric Radiology, Institute of Radiology, St. Luke’s Medical Center, Bonifacio Global City, Taguig City, Philippines; 2 Section of Pediatric Radiology, Institute of Radiology, St. Luke’s Medical Center, Quezon City, Philippines; 3 Department of Paediatric Radiology, Bristol Royal Hospital for Children and the University of Bristol, Bristol, UK; 4 Section of Pediatric Radiology, Clínica de Diagnóstico por Imagem, Rio de Janeiro, Brazil; 5 Section of Pediatric Pulmonology, Institute of Pulmonary Medicine, St. Luke’s Medical Center, Quezon City, Philippines

**Keywords:** Children, Computed tomography, Ghon focus, Progressive primary tuberculosis, Radiography, Tuberculosis

## Abstract

There is a lack of standardized approach and terminology to classify the diverse spectrum of manifestations in tuberculosis. It is important to recognize the different clinical and radiographic patterns to guide treatment. As a result of changing epidemiology, there is considerable overlap in the radiologic presentations of primary tuberculosis and post-primary tuberculosis. In this article we promote a standardized approach in clinical and radiographic classification for children suspected of having or diagnosed with childhood tuberculosis. We propose standardized terms to diminish confusion and miscommunication, which can affect management. In addition, we present pitfalls and limitations of imaging.

## Introduction

Numerous articles have been published regarding childhood tuberculosis but there is still a lack of standardized approach and terminology to classify the diverse spectrum of manifestations in tuberculosis. It is important to recognize the different clinical and radiographic patterns because management of each condition is varied. A classification of disease manifestation that is common to all will help to facilitate communication and understanding among scientific communities.

Generally the pathological changes in childhood tuberculosis are pauci-bacillary, and thus the diagnosis of intrathoracic tuberculosis depends largely on chest imaging [1]. Pulmonary tuberculosis has been classically classified as primary tuberculosis in children and post-primary tuberculosis in adults. However, because of the changing epidemiology there is a considerable overlap in the radiologic presentations of these entities [2].

In this article we promote a standardized combined clinical and radiographic approach for children suspected of having or diagnosed with childhood tuberculosis. We propose standardized terms to diminish confusion and miscommunication, which can affect management. In addition, we present pitfalls and limitations of imaging in childhood tuberculosis diagnosis. The atypical radiologic patterns seen in immunocompromised children, however, are not discussed in this article.

## Clinical categories for intrathoracic tuberculosis in children [3–5]

Intrathoracic tuberculosis has been classified based on clinical, laboratory and radiologic evidence. The child should present at least one sign or symptom suggestive of tuberculosis but without any other plausible etiology. These signs and symptoms include any of (a) persistent cough; (b) weight loss/failure to thrive; (c) persistent unexplained fever, or (d) persistent, unexplained lethargy or reduced activity. The following sections are definitions that have been proposed to indicate the degree of certainty of the diagnosis of tuberculosis.

### Confirmed tuberculosis

Tuberculosis is confirmed when the culture from a specimen representative of intrathoracic disease (e.g., sputum, nasopharyngeal/gastric aspirate, pleural fluid) is positive, and more recently when Xpert MTB/RIF — a rapid test to simultaneously detect *Mycobacterium tuberculosis* and resistance to rifampicin — from any specimen is positive.

The Xpert MTB/RIF is not only sensitive and specific for diagnosing pediatric pulmonary mycobacterial tuberculosis but is also effective in detecting rifampicin resistance [6, 7]. The World Health Organization in 2013 [8] strongly recommended Xpert MTB/RIF for use rather than conventional microscopy, culture and drug-susceptibility testing as the initial diagnostic test in children suspected of having multidrug-resistant-tuberculosis or human immunodeficiency virus (HIV)-associated tuberculosis. This test can also be used (conditional recommendation) rather than conventional microscopy and culture as the initial diagnostic test in all children suspected of having tuberculosis.

### Probable tuberculosis

Children in this category have chest radiographs showing findings consistent with intrathoracic tuberculosis disease, and at least one of the following:

- positive clinical response to anti-tuberculosis therapy,
- documented exposure/close contact with a known tuberculosis patient, or
- positive tuberculin skin test or interferon-gamma release assay.

### Possible tuberculosis

There are two scenarios in this category. One is when the chest radiograph is not consistent with tuberculous disease, but at least one of the criteria in the prior section is present. The other possibility is when the chest radiography is consistent with tuberculosis disease but none of the criteria in the prior section is present.

Some children are symptomatic but have chest radiography findings that are not consistent with tuberculous disease and have none of the criteria mentioned in probable tuberculosis. These children are categorized as either *unlikely tuberculosis* if no alternative diagnosis is established or *not tuberculosis* if an alternative diagnosis is established such as cardiac disease, foreign body aspiration or asthma. Those who have documented exposure or close contact with a known tuberculosis patient but are asymptomatic and have negative tuberculin skin test and chest radiography are considered *tuberculosis exposed*. No treatment is necessary for these children.

Tuberculous infection and tuberculous disease have to be differentiated because treatments for these two entities are different. We propose simple definitions. When a child has a positive tuberculin skin test but does not show any of the probable tuberculosis symptoms and has a normal chest radiograph, this might be classified as tuberculous infection and could be treated with one-drug therapy [9]. However when the findings in the chest radiographs are consistent with tuberculosis, this is considered tuberculous disease and warrants treatment with at least three drugs [9–11]. In the next sections we discuss chest radiography findings that are consistent with tuberculous disease seen in possible or probable tuberculosis.

## Primary pulmonary tuberculous disease

**Fig. 1:**
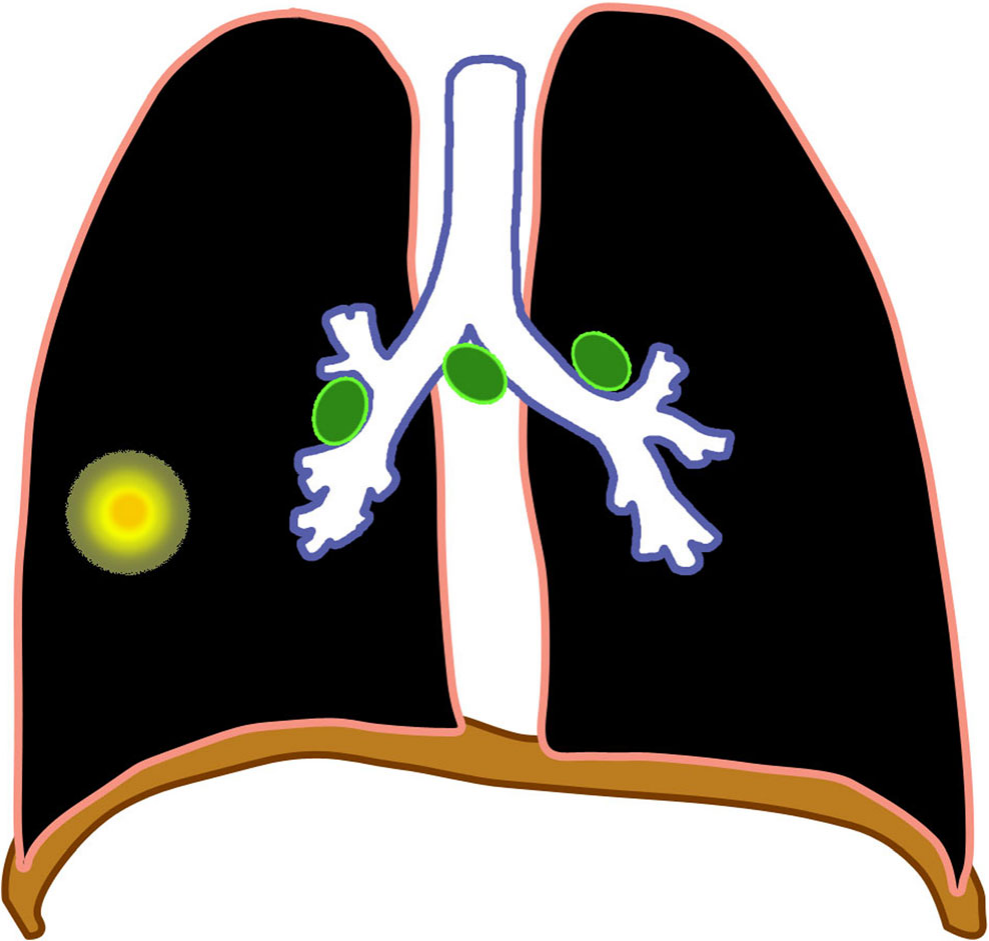
Diagram of a Ghon focus (*yellow*) and associated lymphadenopathy (*green*). This is the so-called primary complex

**Fig. 2:**
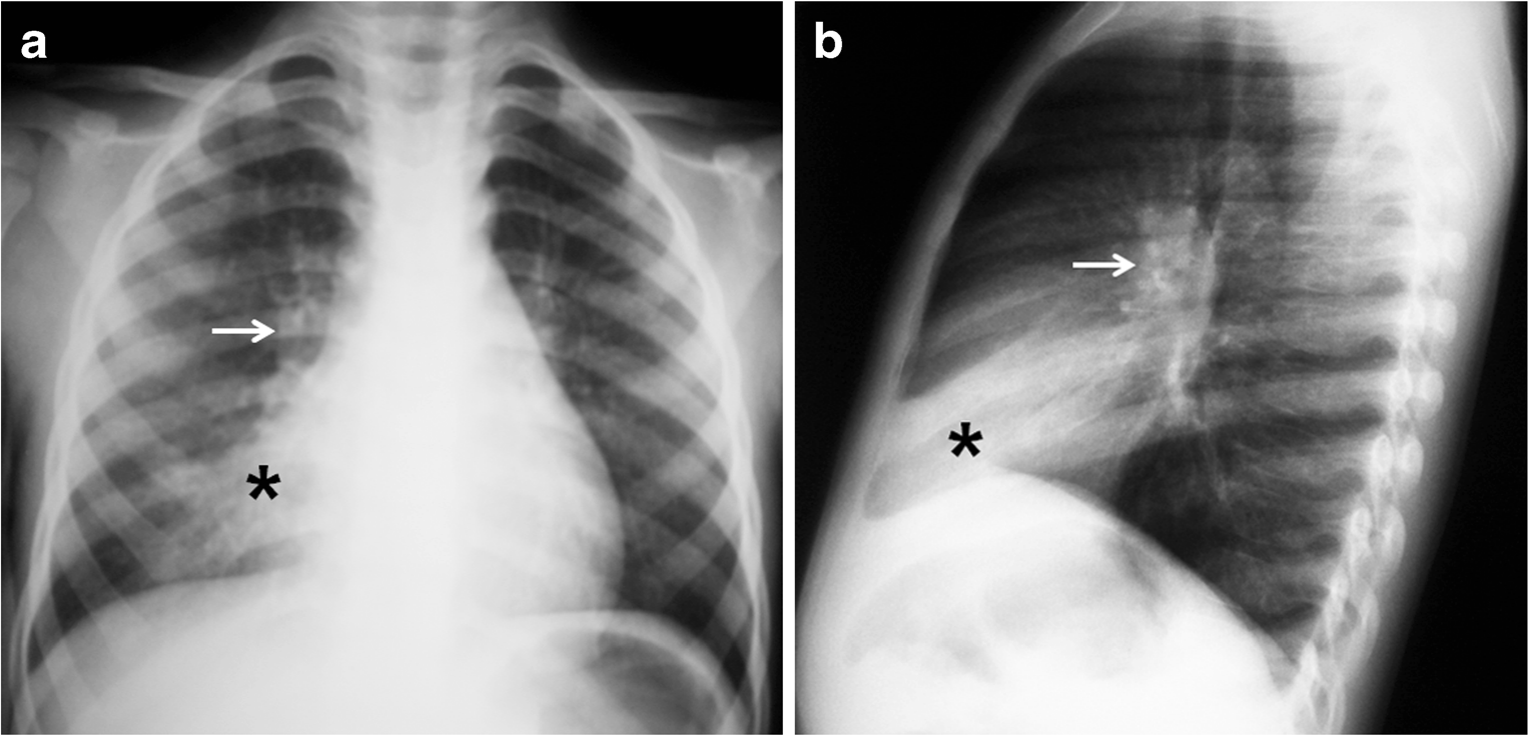
Chest radiographs in a 5-year-old girl with primary tuberculous disease. **a, b** Anteroposterior (**a**) and lateral (**b**) views show a middle lobe opacity (*asterisk*) with right hilar lymphadenopathy (*arrow*)

The major route of *Mycobacterium tuberculosis* infection is by inhalation [1]. Infection begins when infected droplets are deposited in the terminal airway or alveoli, followed by a localized parenchymal inflammation or pneumonic process called the primary (Ghon) focus. There is then spread via draining lymphatic vessels, usually to the ipsilateral central or regional lymph nodes, which then enlarge. The upper lobes drain to the ipsilateral paratracheal nodes, while the rest of the lung drains to the perihilar nodes. The parenchymal focus and the enlarged lymph nodes are called the primary (Ranke or Ghon) complex [1, 2, 12–17] (Figs. 1 and 2).

Incubation can be up to 6 weeks from exposure, during which time chest radiographs are normal. After 1–3 months from exposure, hilar or mediastinal adenopathy can be visualized in 50–70% of cases [18–20]. Primary tuberculosis reflects a patient’s conversion from insensitivity to having the antigens of the tubercle bacilli [2, 14].

**Fig. 3:**
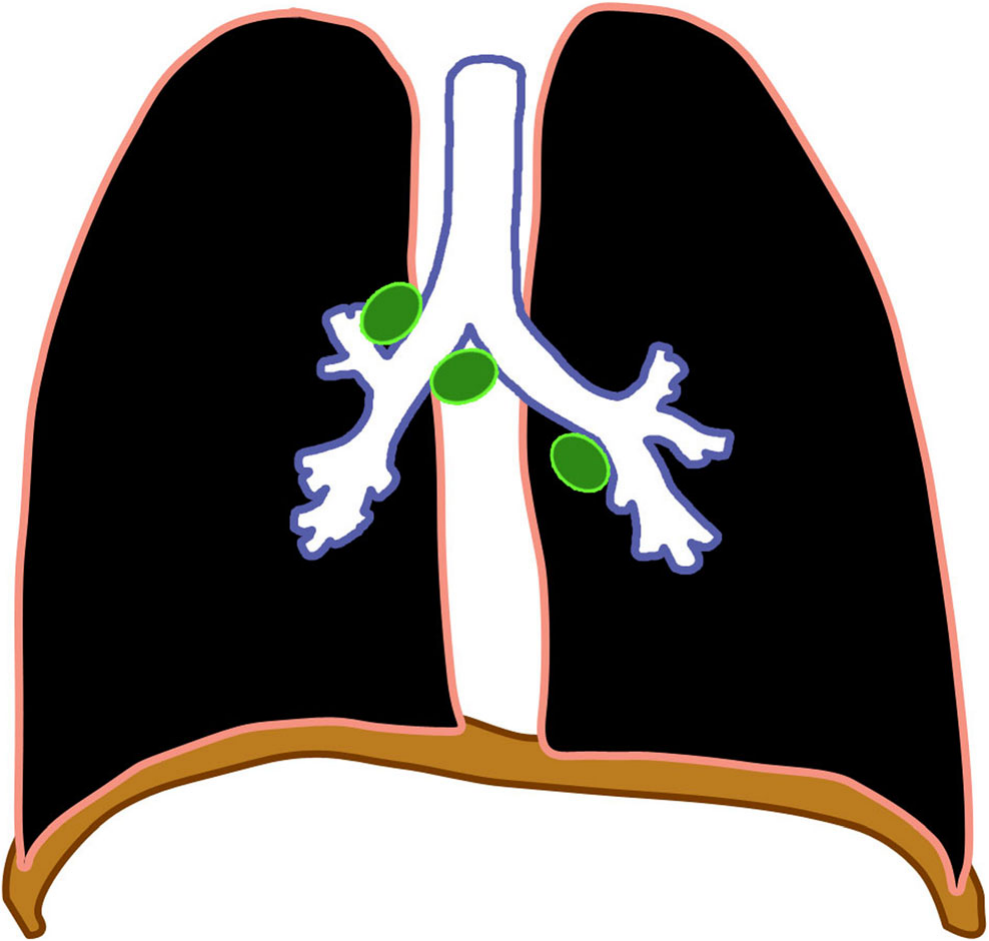
Diagram of isolated lymphadenopathy (*green*)

**Fig. 4:**
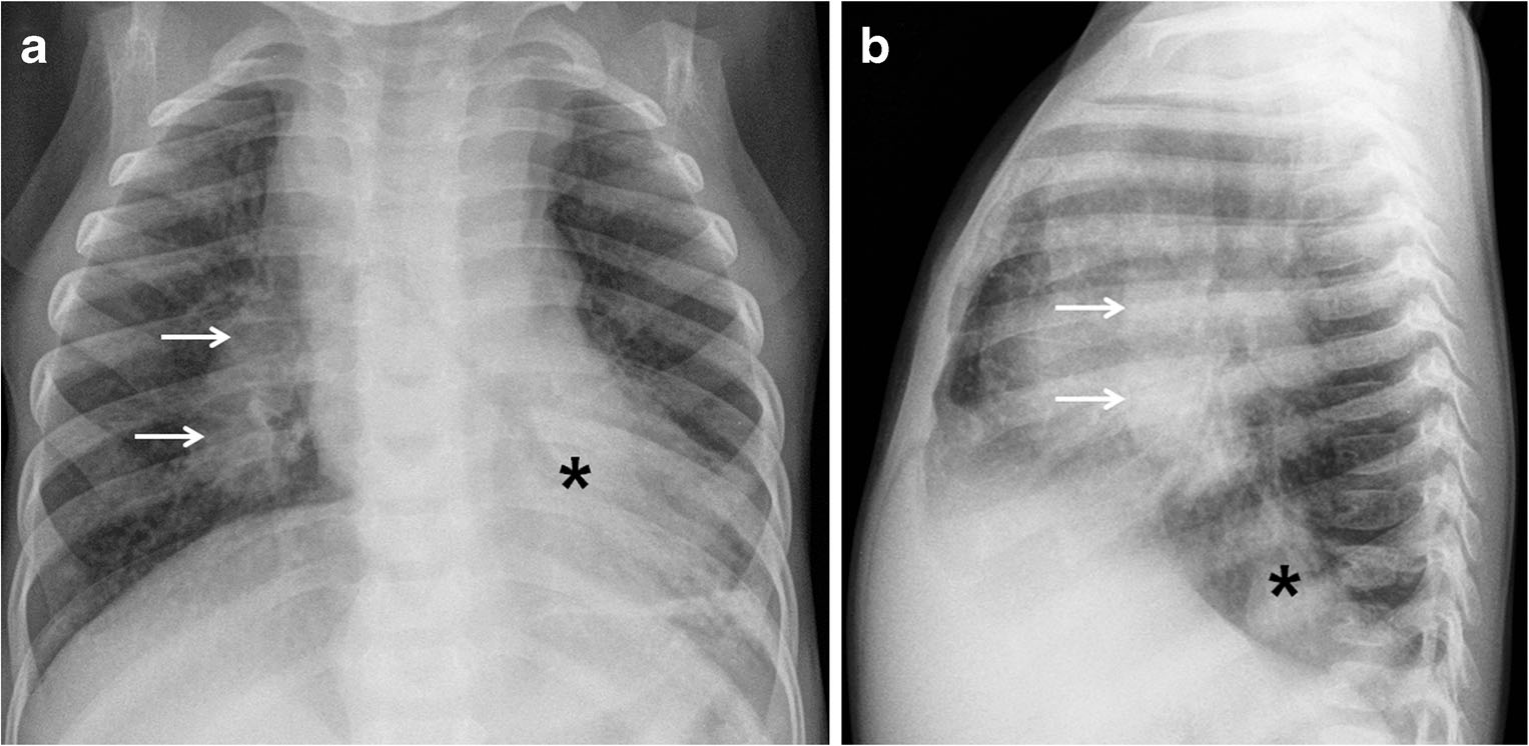
Chest radiographs in a 1-year-old boy with primary tuberculous disease and lymphadenopathy. **a, b** Anteroposterior (**a**) and lateral (**b**) views show hilar lymphadenopathy (*arrows*) on the right without ipsilateral lung abnormality. A left retrocardiac opacity (*asterisk*) is noted

Regional (perihilar or paratracheal) lymphadenopathy is the radiologic hallmark of primary infection in childhood [1, 13] (Figs. 3 and 4). Anteroposterior and lateral views are required for optimal lymph node visualization [13, 21], but it can remain difficult to visualize enlarged lymph nodes with certainty [1, 22]. The most common sites of nodal involvement are the right paratracheal and hilar regions [13, 17].

The prevalence of adenopathy decreases with age; it is 100% in children <3 years of age and 88% in older children. The prevalence of parenchymal involvement detectable on radiographs, however, is significantly lower in children <3 years of age (51%) as compared with that in older children (78%) [13].

**Fig. 5:**
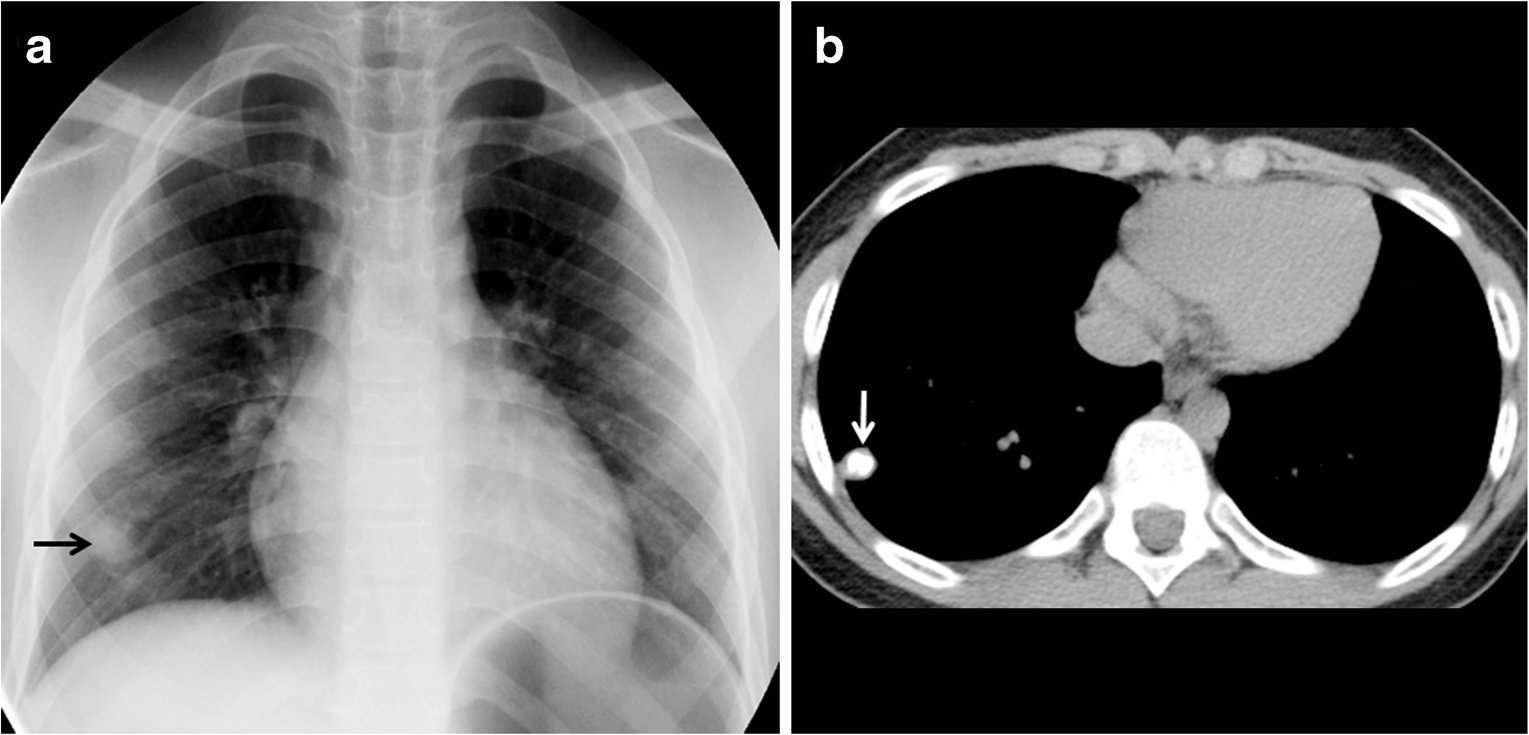
Ghon focus in a 7-year-old boy. **a** Anteroposterior chest radiograph shows a Ghon focus (*arrow*) in the right lower lobe. **b** Non-enhanced axial chest CT demonstrates the focus, which is calcified (*arrow*)

If the child is immunocompetent, the lesions heal and become dormant while still causing continuous antigenic stimulation for maintenance of hypersensitivity to tuberculous antigen. Thus the tuberculin skin test is positive in 95% of cases. This has been referred to as latent tuberculous infection. The caseating necrosis within the Ghon focus and infected lymph node frequently calcifies [1, 2]. Calcification can occur from 6 months to 4 years after infection, occurring earlier in young children [1]. The parenchymal focus, called pulmonary tuberculomas (Fig. 5), which are identified radiographically, represent sharply defined ovoid granulomas, solitary or multiple, ranging in size from 0.4 cm to 5 cm in diameter [2, 23]. Children with latent tuberculous infection can be treated with a one-drug therapy provided there was no prior treatment.

If immunity is inadequate, the disease progresses either locally or in other parts of the lung or body, with spread of infection via the airways, lymphatics or bloodstream [15, 16]. Clinically active tuberculous disease can develop within 5 years after infection. This is called progressive primary tuberculosis [2, 24].

## Progressive primary tuberculous disease

Progression from infection to disease usually occurs within 1 year after the primary infection in more than 90% of cases. It is bimodal in age distribution, with children younger than 5 years and adolescents being at increased risk [1, 17, 25].

**Fig. 6:**
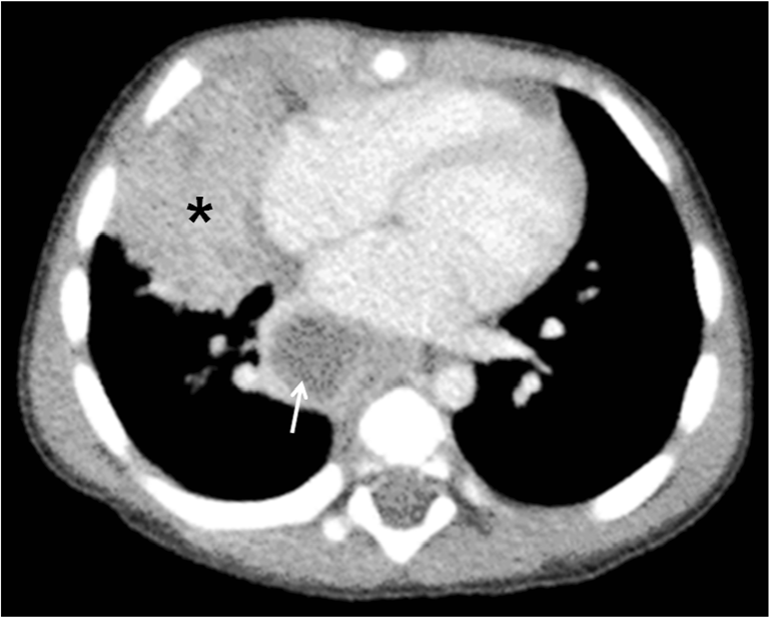
Primary progressive tuberculous disease in a 15-month-old boy. Axial contrast-enhanced CT image shows progressive Ghon focus (*asterisk*) in the right middle lobe and progressive hilar lymphadenopathy (*arrow*). The enlarged lymph node demonstrates the characteristic central necrosis with peripheral enhancement (rim sign)

**Fig. 7:**
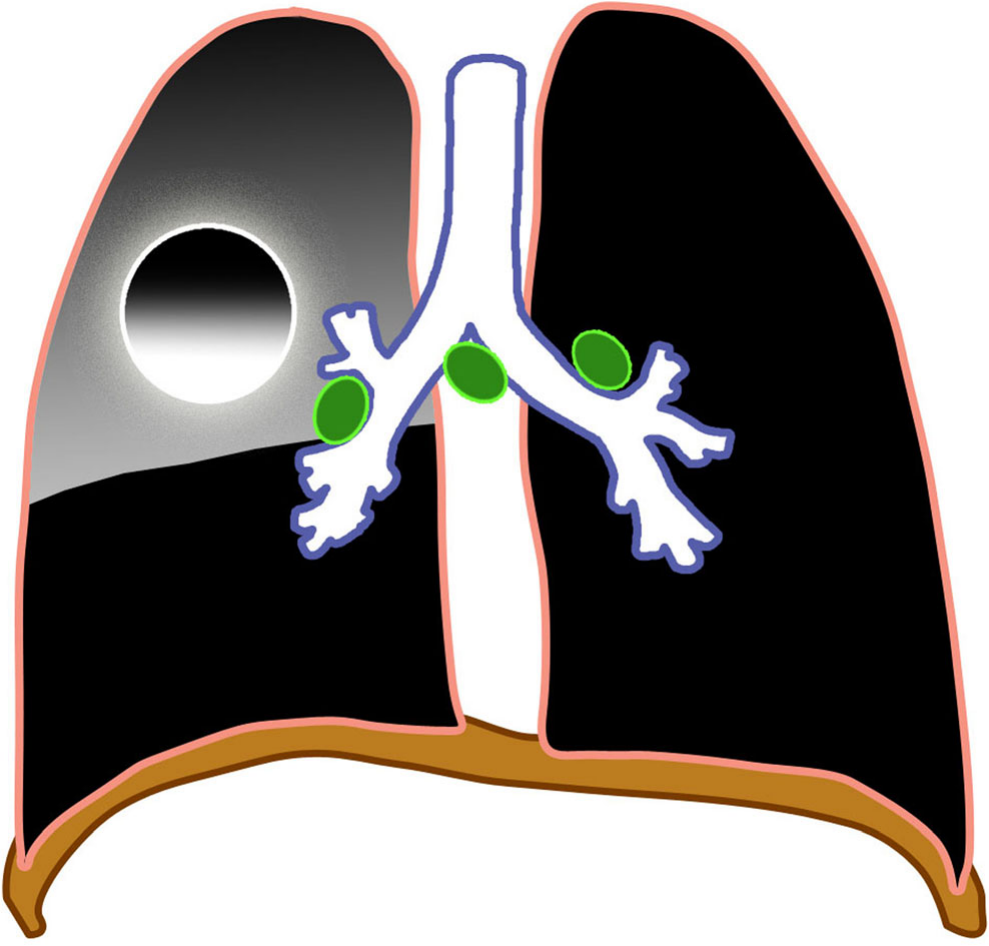
Diagram shows a progressive Ghon focus (*circle*) with cavitation and associated lobar consolidation

Early disease progression can happen 2–6 months from exposure, when homogeneous consolidation can occur (Fig. 6). Obstructive atelectasis or overinflation can result from compression by an adjacent enlarged node. Distribution is typically on the right side at the level of the right lobar bronchus or bronchus intermedius. Fibrosis and destruction of the lung parenchyma result in traction bronchiectasis and formation of cavities (Fig. 7), respectively [2]. This is known as progressive Ghon focus [18–20].

Three possible mechanisms are involved in the formation of cavities: (1) progressive primary spread of disease with extensive and bilateral pulmonary cavities, (2) cavities caused by bronchial obstruction by lymph nodes or (3) post-primary tuberculosis showing cavities that are usually single and unilateral in the upper lobe. These have fairly equal incidence [26].

Lymph nodes can continue to enlarge 4–12 months from exposure and can cause progression of the disease affecting the airways, pleura and pericardium, which are discussed in the next sections. On contrast-enhanced CT, involved lymph nodes often measure more than 2 cm and show a very characteristic, but not pathognomonic, rim sign consisting of a low-density center surrounded by a peripheral enhancing rim [17, 22, 27] (Fig. 6). The esophagus, lymphatic duct and phrenic nerve might also be affected, producing tracheoesophageal fistula, chylothorax and diaphragmatic palsy, respectively [1, 18–20]. Hematogenous miliary dissemination can also occur in this stage.

### Miliary tuberculosis

**Fig. 8:**
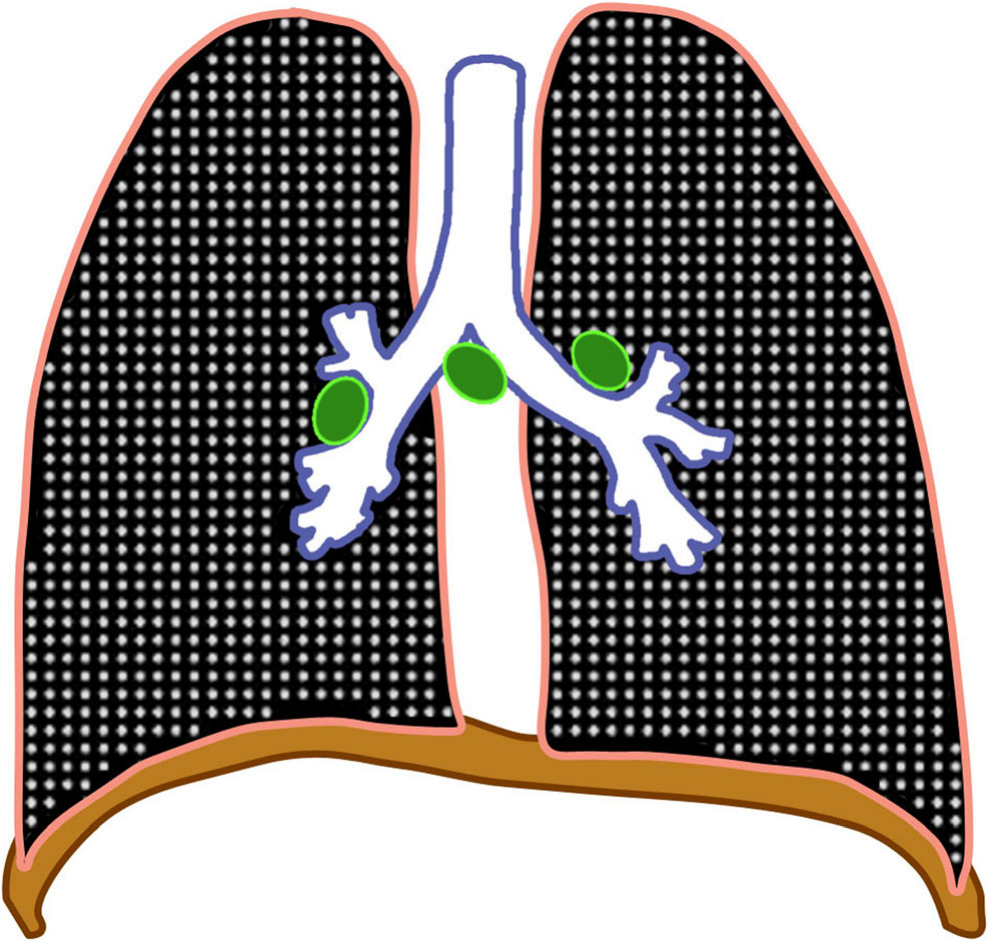
Diagram of miliary tuberculosis (*white dots*) with lymphadenopathy (*green*)

**Fig. 9:**
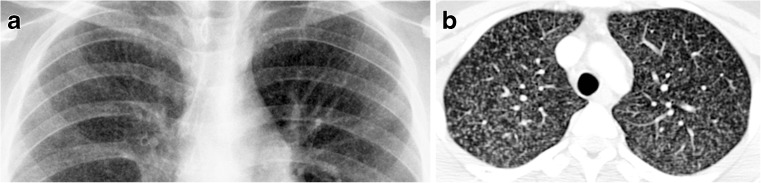
Miliary tuberculosis in a 14-year-old boy. **a, b** Focused anteroposterior chest radiograph (**a**) and axial contrast-enhanced CT (**b**) of the upper lobes show innumerable 2-mm or smaller discrete nodules

Miliary tuberculosis is seen in 8% of cases [25], usually in the younger age group because of immature immune function [28]. It is an acute hematogenously disseminated infection presenting as innumerable ≤2-mm non-calcified nodules scattered in both lungs [1, 2, 18–20, 28–30] (Figs. 8 and 9). There is no pathognomonic finding for tuberculosis except for miliary tuberculosis [28], and it can be seen in primary and post-primary disease [2].

In 25–40% cases, chest radiographs are initially normal [1, 30]. CT is more sensitive for miliary disease before it becomes radiographically apparent. The tiny nodules can be sharply or poorly defined, and are seen in a diffuse, random distribution, often with intra- and interlobular septal thickening [16].

### Lymphobronchial/lymphotracheobronchial tuberculosis

**Fig. 10:**
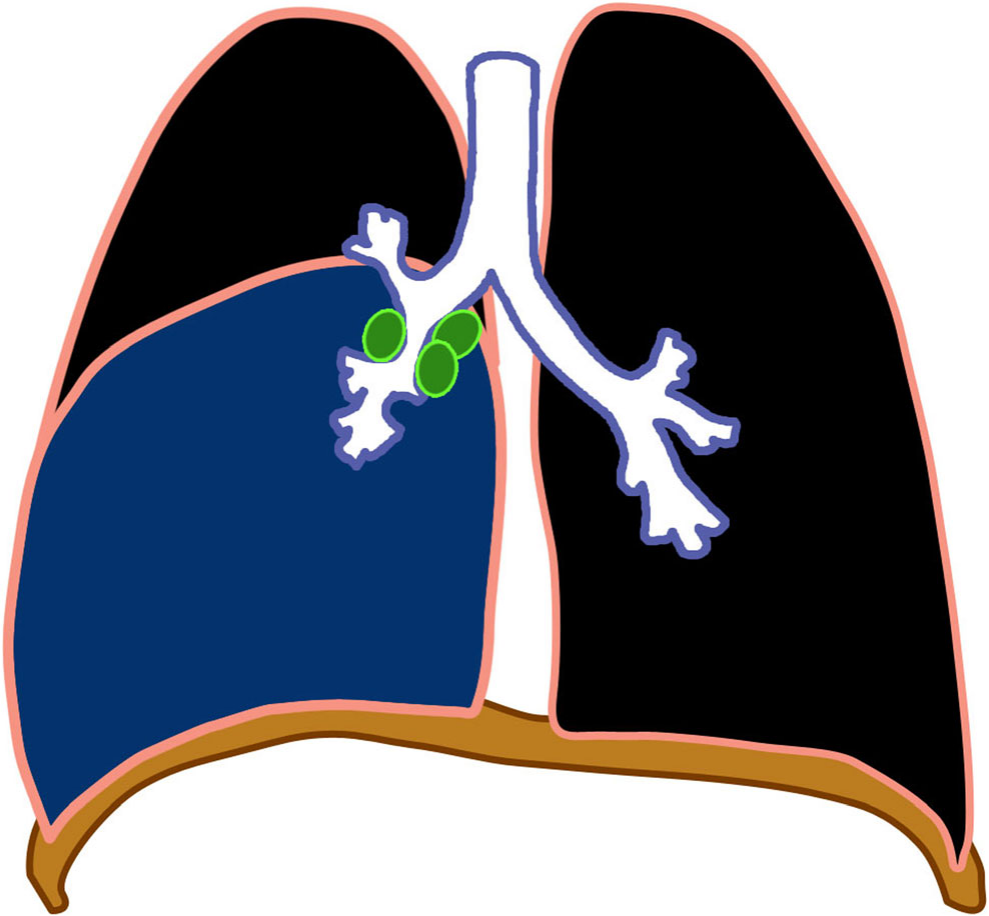
Diagram of lymphobronchial tuberculosis with partial obstruction of the lower lobe bronchus by enlarged lymph nodes (*green*) and secondary lobar hyperinflation (*blue*)

**Fig. 11:**
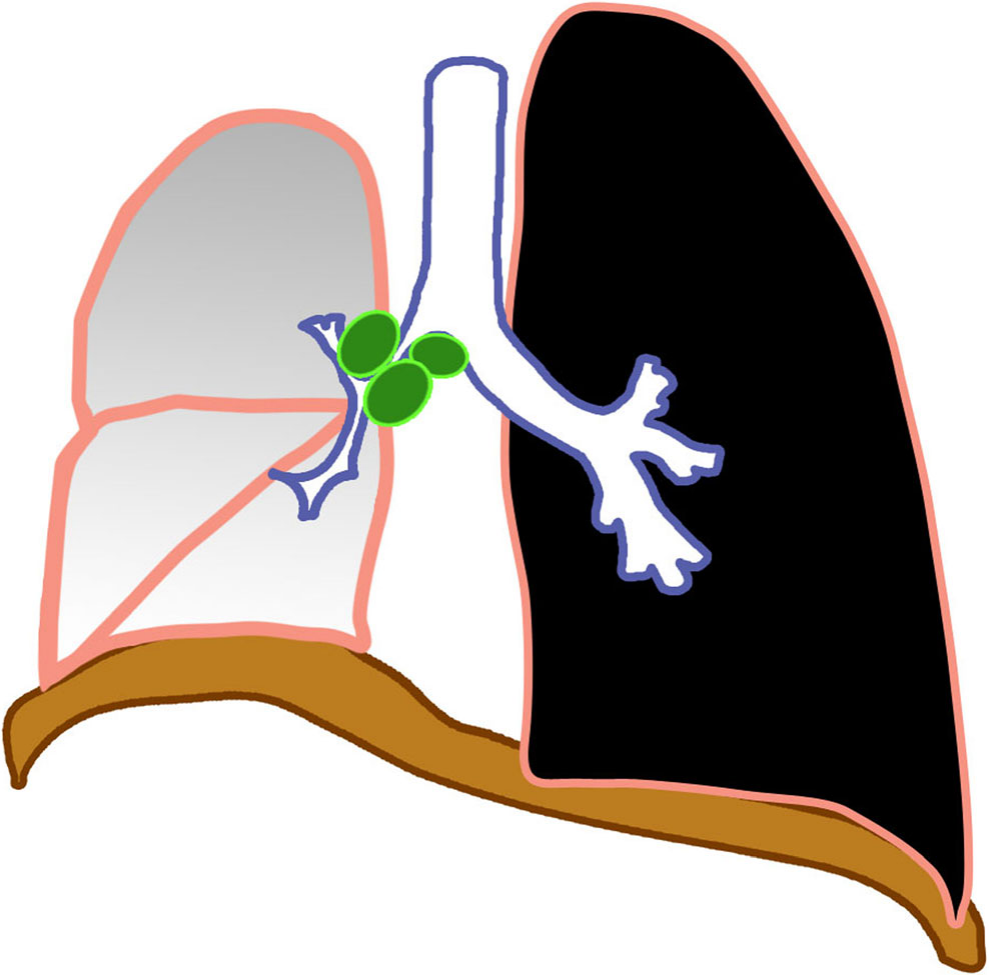
Diagram of lymphobronchial tuberculosis with complete obstruction by enlarged lymph nodes (*green*) of the right main bronchus, resulting in right lung atelectasis

**Fig. 12:**
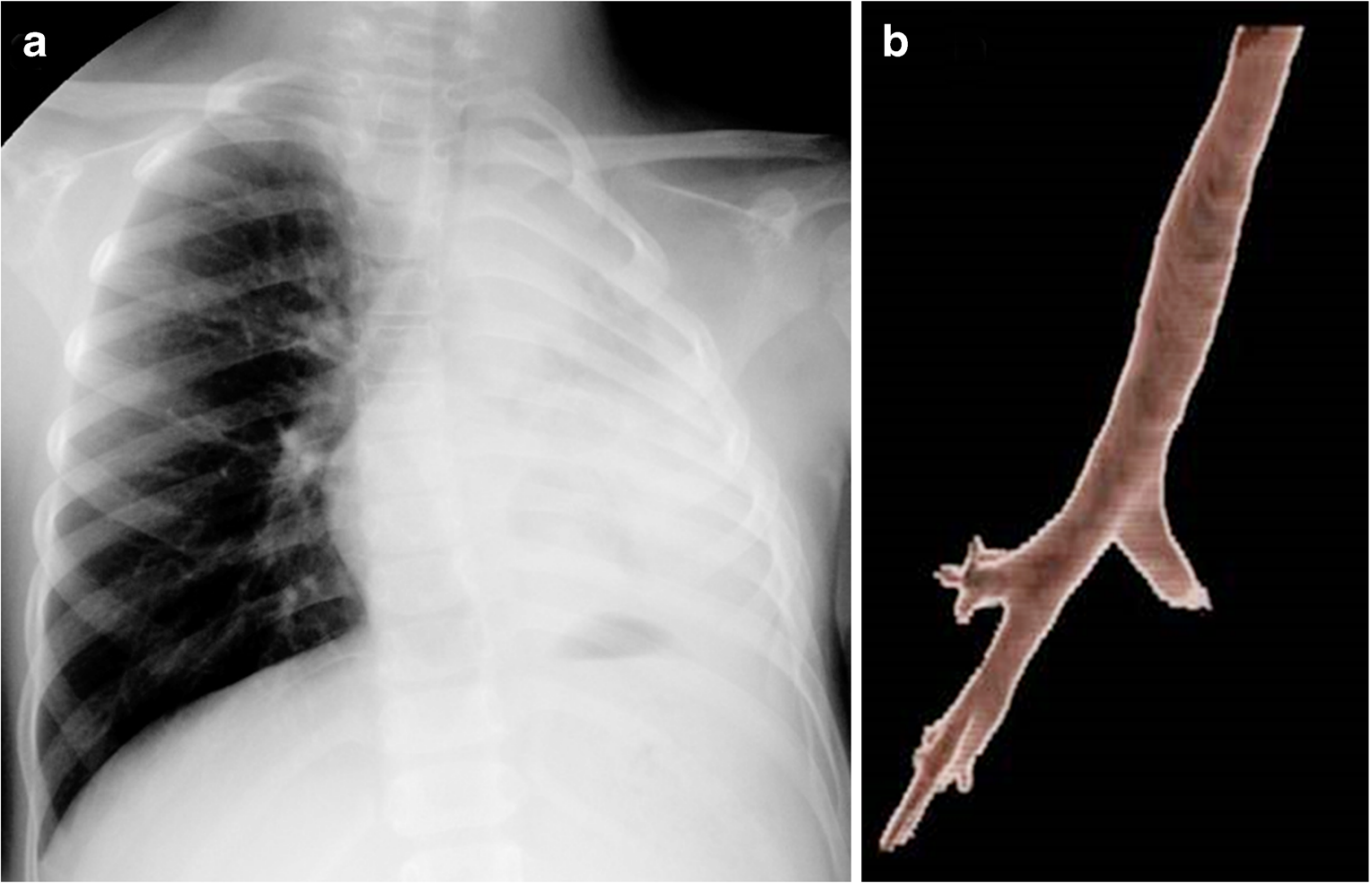
Primary progressive lymphobronchial tuberculous disease in a 6-year-old boy. **a** Anteroposterior chest radiograph shows left lung collapse. **b** Volume-rendered CT shows occlusion of the left mainstem bronchus caused by lymphobronchial disease

Lymphobronchial or lymphotracheobronchial involvement is a complication in 2–4% of tuberculosis cases [2, 31]. Lymphadenopathy is seen in chest radiographs in 63–95% [26] and on CT in up to 96–100% of tracheobronchial tuberculosis cases [32, 33]. The enlarged nodes compress the adjacent trachea or bronchi, causing luminal narrowing and resulting in lung hyperinflation from partial obstruction with check-valve effect (Fig. 10), or atelectasis due to a complete obstruction (Figs. 11 and 12). These nodes subsequently erode, perforate and discharge caseous material into the airways manifesting as obstructive pneumonia [1, 2, 13, 14, 17–20, 33–36]. Lymphogenic and hematogenous spread into the large airways have also been reported [2].

Radiographic manifestations in lymphotracheobronchial tuberculosis are nonspecific, and a normal chest radiograph does not rule out airway involvement. Involvement of the central airways can be easily missed on radiographs. Persistent segmental or lobar collapse, lobar hyperinflation and obstructive pneumonia are seen as complications of the airway compression [31, 33].

Enhancement and enlargement (usually >2 cm) of adjacent mediastinal lymph nodes are common findings at CT in the active stage of stenosis. The enlarged lymph nodes are commonly identified in the subcarinal [37], paratracheal and perihilar (infrahilar) regions closely abutting or compressing the airways [33].

The most commonly involved airway is the bronchus intermedius, followed by the left main bronchus and trachea [37]. Bronchial narrowing can be smooth or irregular, with mural thickening [33, 38]. Smooth bronchial narrowing is caused by compression by an adjacent node, and the irregular narrowing correlates with significant mucosal irregularity, caseation, granuloma formation or even perforation [33].

**Fig. 13:**
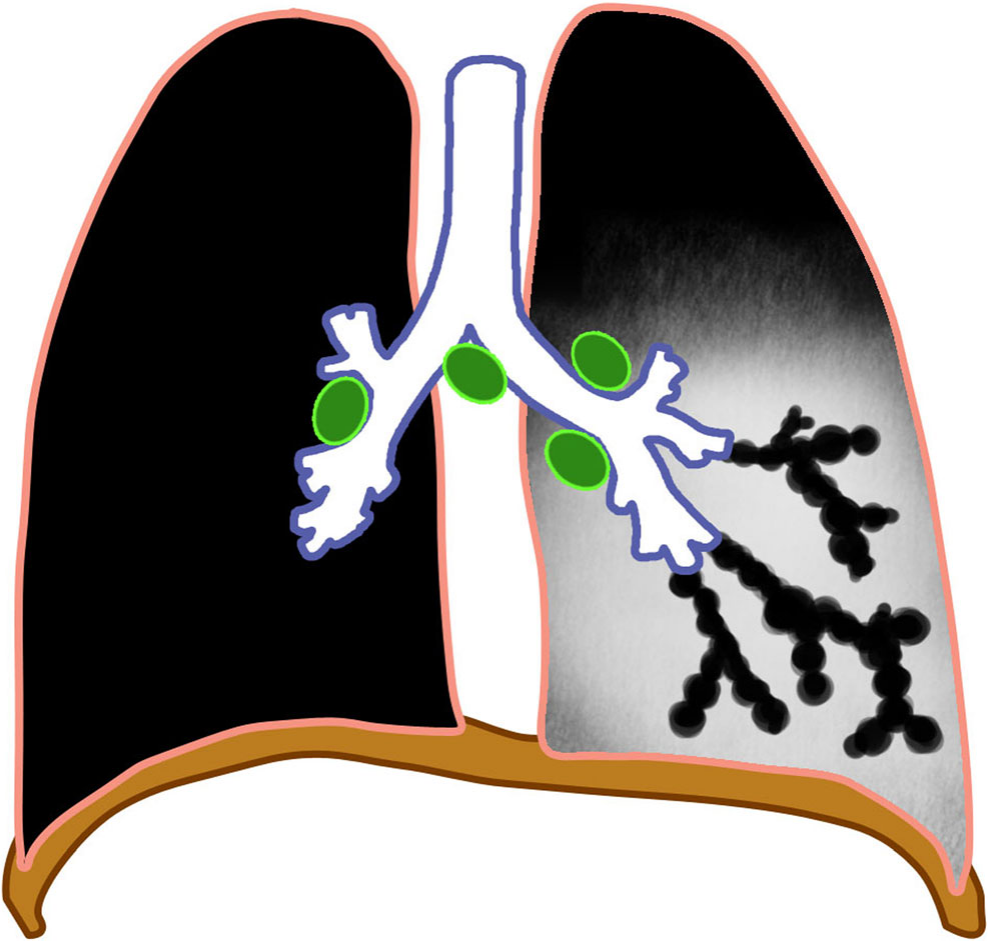
Diagram of bronchiectasis (*beaded black lumina*) as a complication of tuberculosis Note enlarged lymph nodes (*green*)

The obstructive infiltrates can be resorbed or calcify, fibrose with traction bronchiectasis (Fig. 13) or cause lung destruction [2]. There is often excessive inflammation, which can result in dense alveolar consolidation and eventual parenchymal breakdown [1]. Cicatricial bronchostenosis can manifest as concentric narrowing, uniform wall thickening, and involvement of a long bronchial segment after healing [31].

### Pleural tuberculous disease

**Fig. 14:**
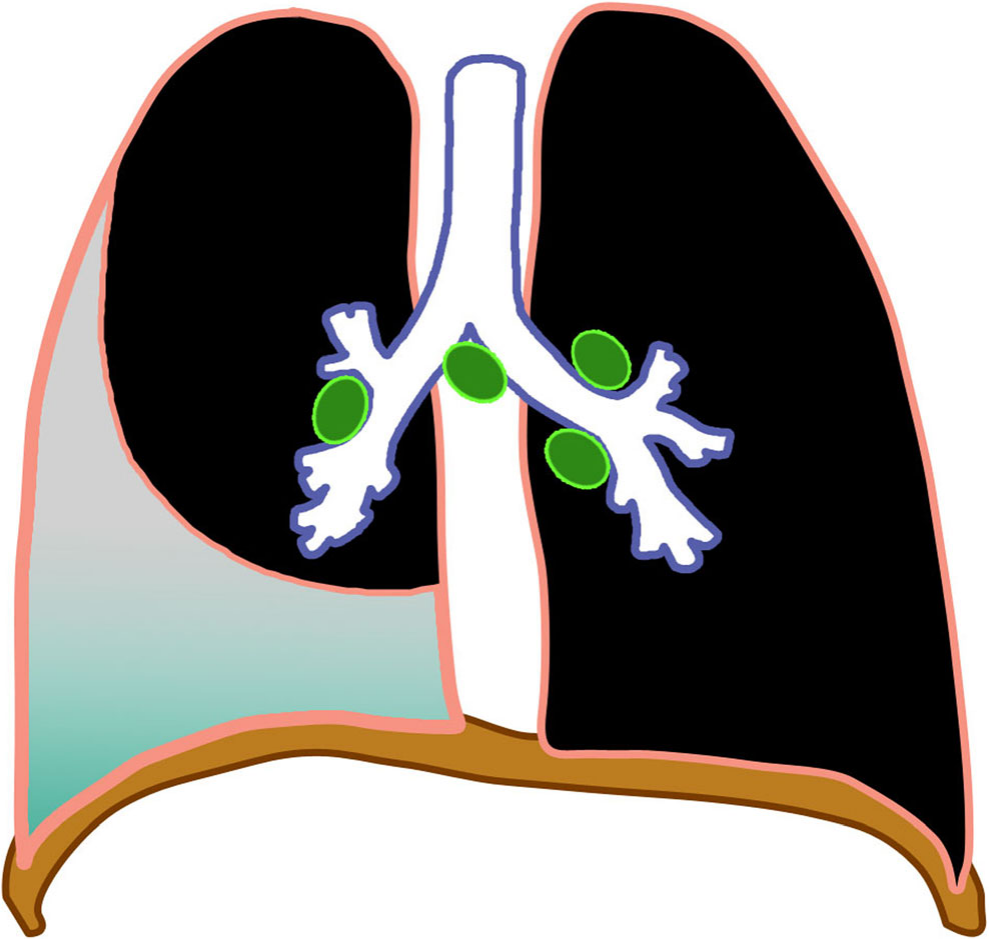
Diagram of pleural effusion as a complication of tuberculosis

**Fig. 15:**
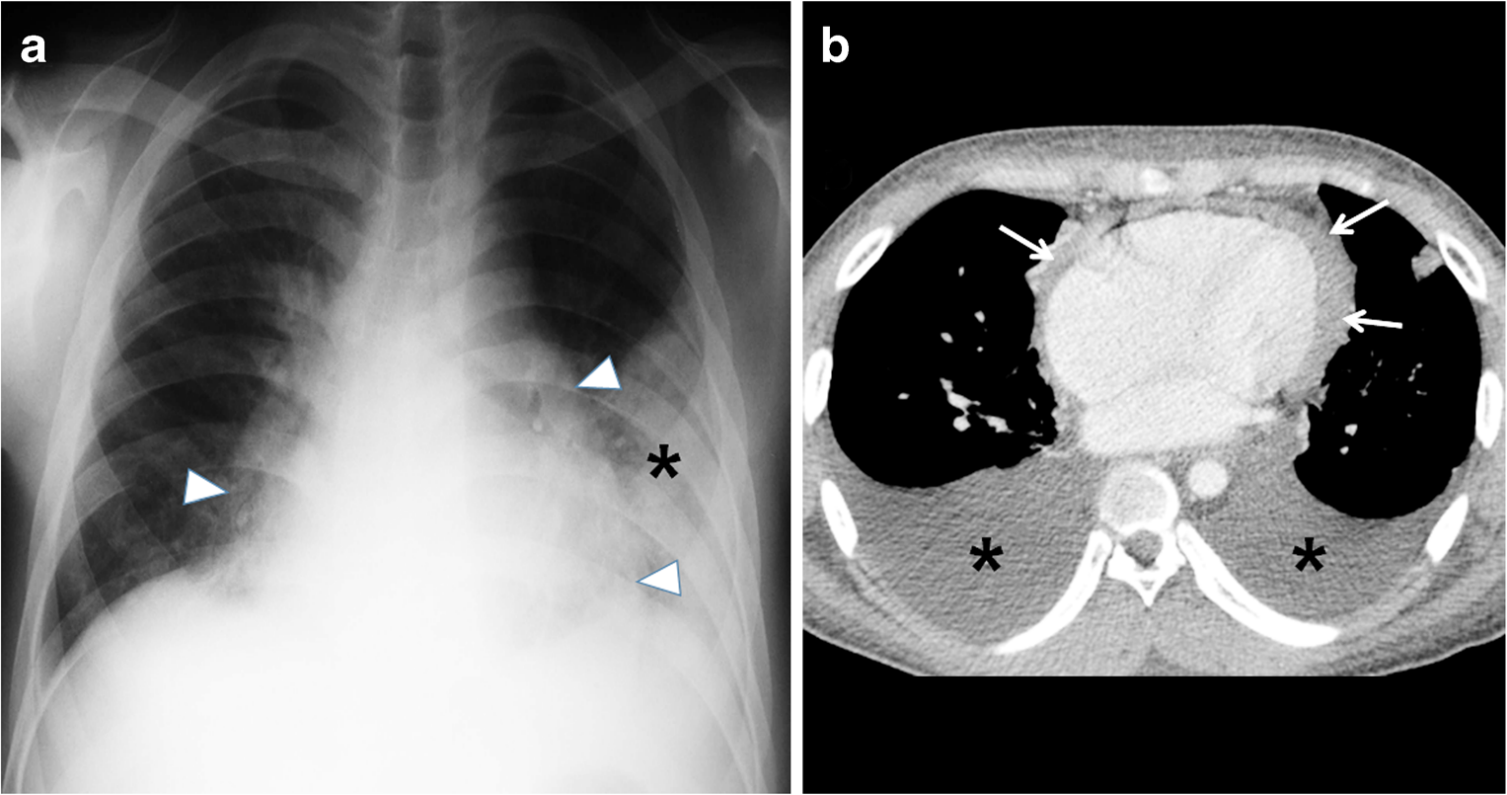
Pleural effusion and cardiac involvement in a 10-year-old girl with primary progressive tuberculous disease. **a** Posteroanterior chest radiograph shows an enlarged cardiac shadow (*arrowheads*) with pleural effusion (*asterisk*) on the left. **b** Follow-up axial contrast-enhanced CT image demonstrates bilateral pleural effusions (*asterisks*) and pericardial effusion (*arrows*)

Another site of extrapulmonary involvement, aside from lymph nodes, is the pleurae [25]. Its prevalence increases with age. Pleural effusion (Figs. 14 and 15) most often results from obstruction of the lymphatic drainage or hypersensitivity reaction than from direct seeding into the pleura. This explains why pleural fluid cultures are mostly negative [28]. Spread to the pleura might also come from a caseating granuloma near the pleura or via hematogenous dissemination [2].

As a complication of primary tuberculosis, pleural involvement is most frequently observed in older children and adolescents. It can occur 3–6 months after infection and is sometimes asymptomatic [2]. It is also typically appreciated in association with parenchymal or nodal disease [39]. Pleural effusions are associated with air-space consolidation in 29% and could be bilateral or loculated in 6% of cases [17]. This is usually self-limiting and prognosis is good. Residual pleural calcifications appear in some cases [2].

The effusion can complicate into an exudative effusion, empyema or infiltration of the thoracic duct [18–20]. Contrast-enhanced CT scan shows smooth thickening of visceral and parietal pleura (“split-pleura” sign) [40]. An Air-fluid level in the pleural space indicates presence of bronchopleural fistula [41]. The empyema can also spread beyond the parietal pleura to produce a subcutaneous abscess, called empyema necessitatis [42].

### Pericardial disease

**Fig. 16:**
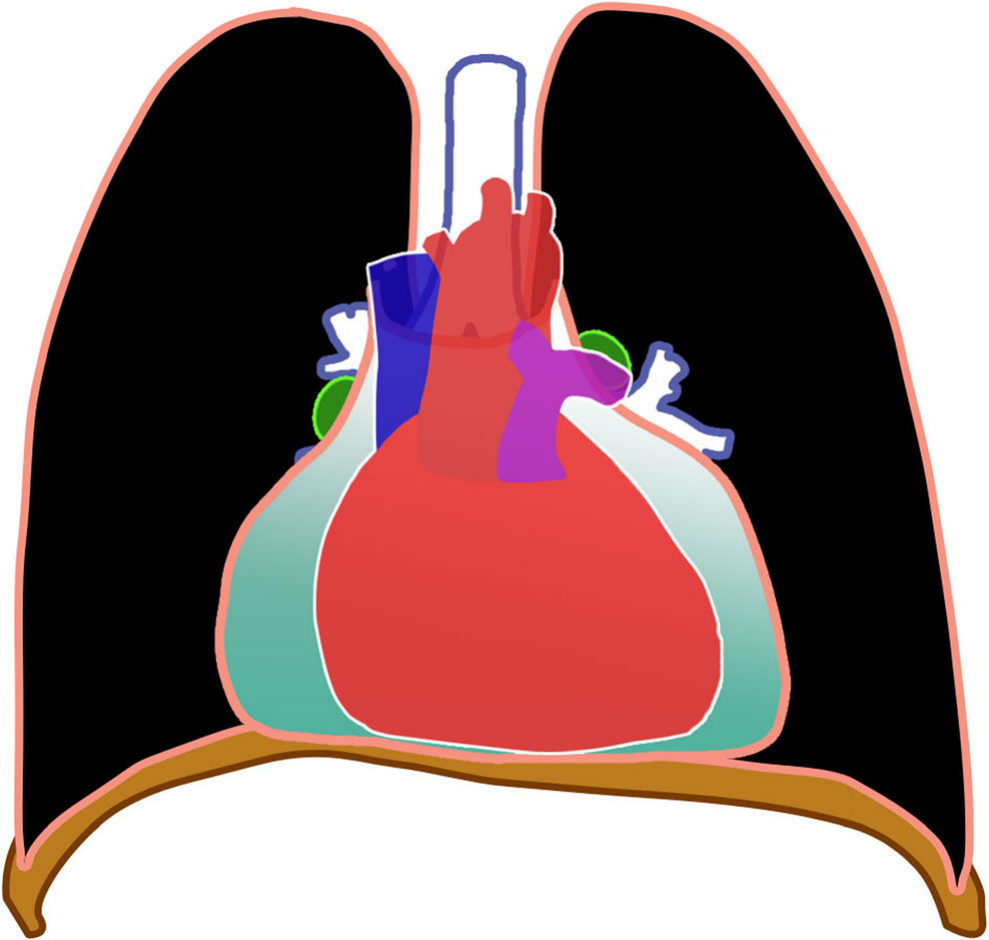
Diagram of pericardial effusion (*green*) as a complication of tuberculosis

**Fig. 17:**
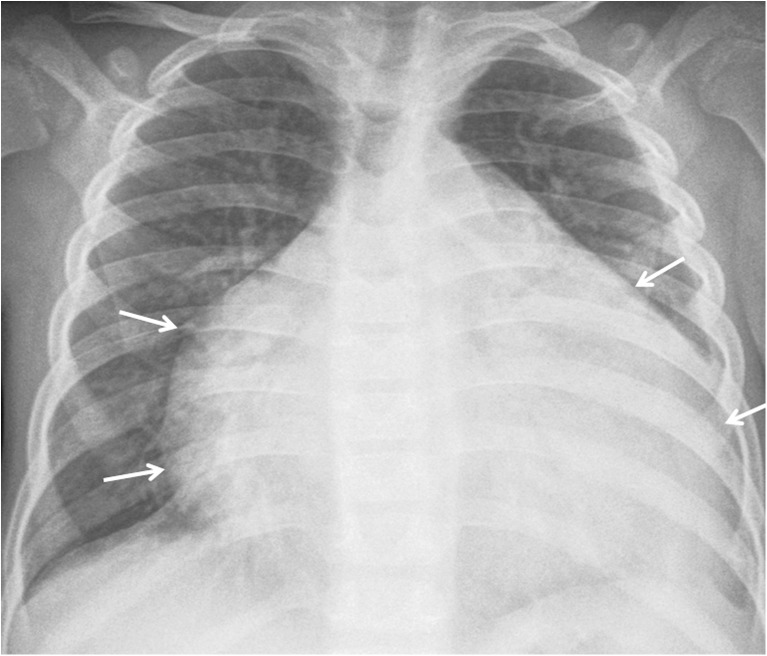
Tuberculous pericarditis in a 6-year-old boy. Anteroposterior radiograph of the chest shows globular enlargement (*arrows*) of the cardiac silhouette (water bottle sign)

Tuberculous pericarditis is a relatively uncommon complication of primary tuberculosis. It has been reported in 1% of cases. It is commonly caused by direct extension of lymph nodes into the posterior pericardial sac [28], although miliary spread has been reported [28, 43]. CT shows lymphadenopathy and pericardial thickening with or without effusion. Constrictive pericarditis with fibrous or calcified pericardial thickening of usually >3 mm occur in about 10% of patients [43]. Pericardial effusion (Figs. 15, 16 and 17), commonly serous type [28], can result in globular enlargement of the heart shadow (water bottle sign) [1].

## Post-primary tuberculosis

Post-primary tuberculosis is also known as adult-type, reactivation or secondary tuberculosis and sometimes phthisis [2]. This results from the reactivation of dormant foci. It is further observed in the pediatric age group, mostly in adolescents [2, 13]. It is considered a late disease progression of the primary infection, which can occur 8–24 months from exposure and in children as young as 8 years [18–20].

**Fig. 18:**
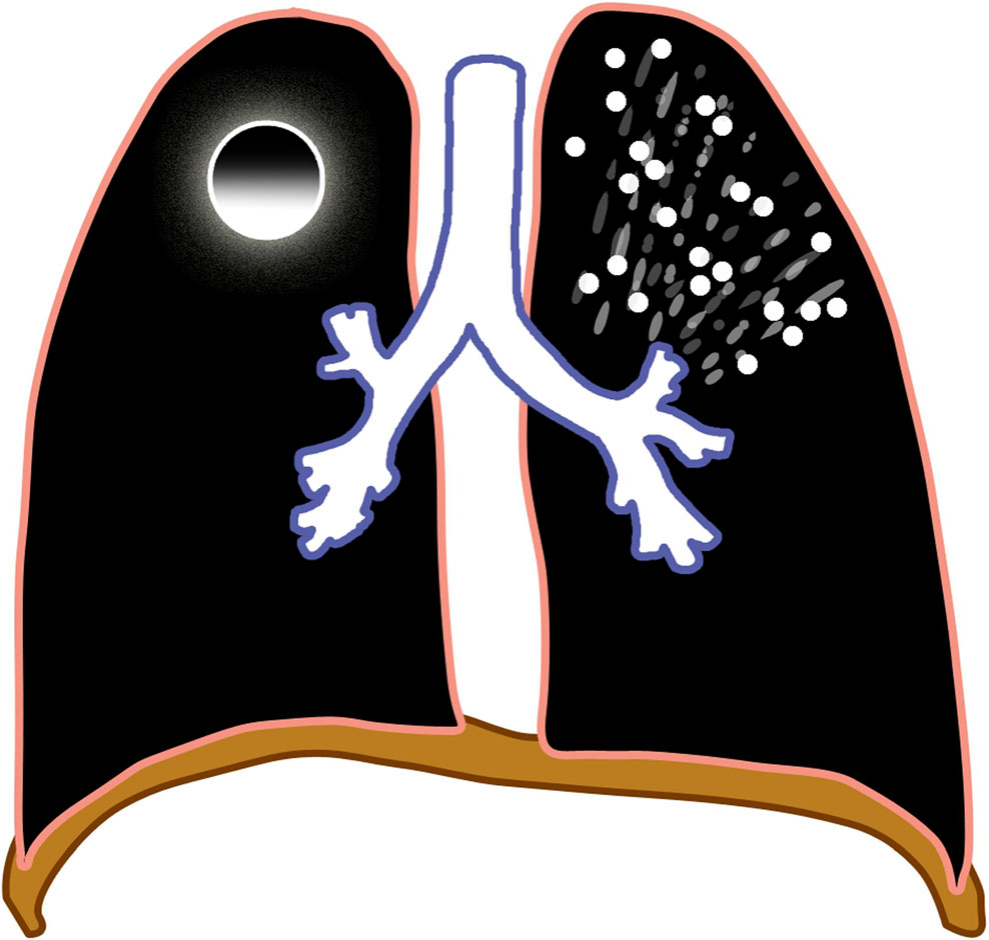
Diagram of a post-primary tuberculosis with upper lobe predominance

The most commonly affected locations are the apical and posterior segments of the upper lobes and the apical segment of the lower lobes because of higher oxygen tension (Fig. 18). Initially there might be cloudy opacification in a segment before coalescence and parenchymal breakdown. Complications include cavitation, bronchogenic spread with bronchopneumonic consolidation, exudative pleuritis, cicatrization atelectasis of the upper lobe with retraction of hilum and formation of traction bronchiectasis [1, 2, 44–46]. Lymph node enlargement is not common in comparison to primary tuberculosis [18–20].

**Fig. 19:**
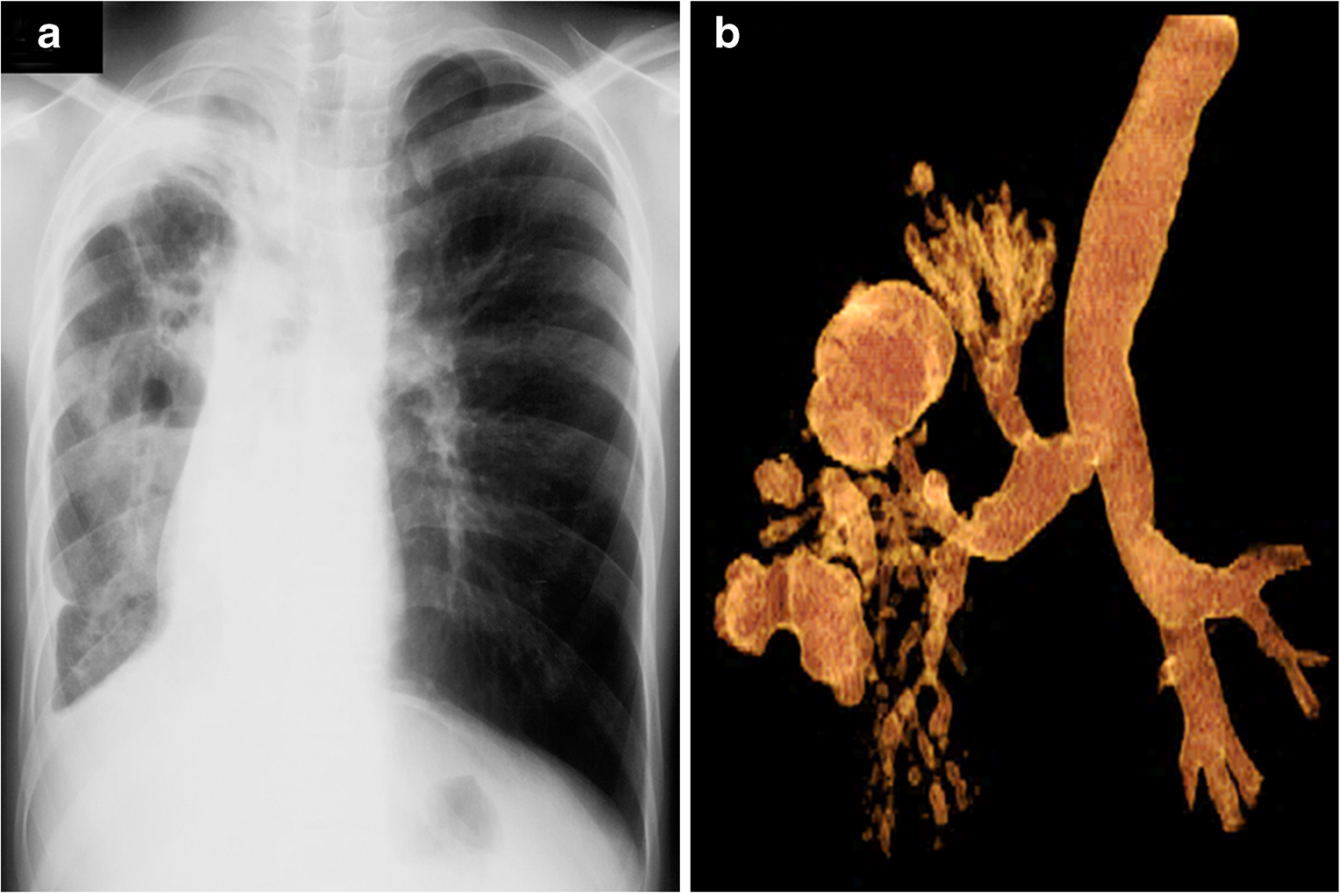
Post-primary tuberculosis in a 14-year-old girl. **a, b** Posteroanterior chest radiograph (**a**) and volume-rendered CT (**b**) show cavitations, traction and cystic bronchiectasis in the right lung

Cavitation is radiographically evident in 40% of cases of post-primary disease. The walls of the cavities might appear thin and smooth or thick and nodular. It is difficult to distinguish thin-walled cavities from bullae, cysts or pneumatoceles. Cystic bronchiectasis should also be considered when multiple cavities are present [47] (Figs. 18 and 19).

**Fig. 20:**
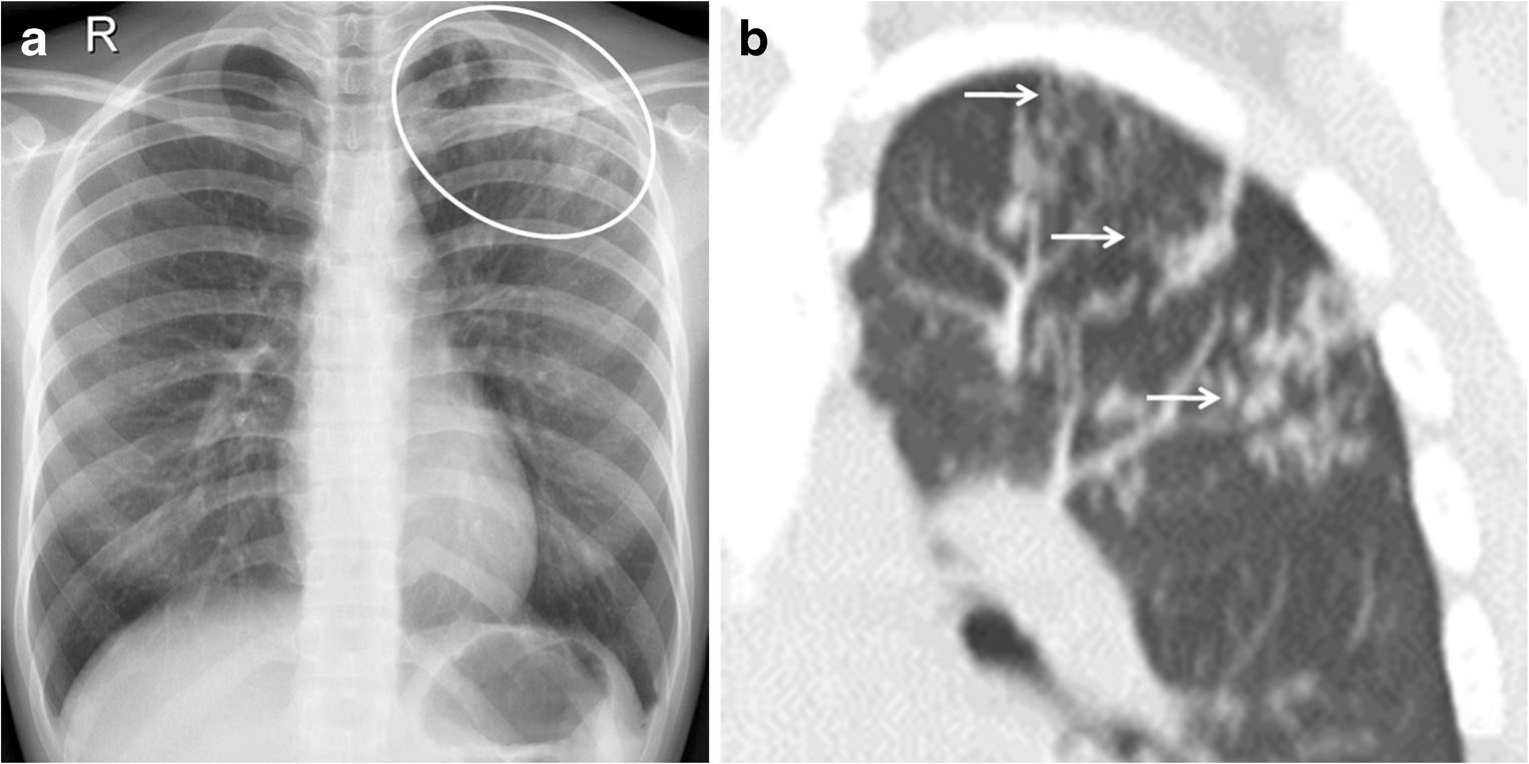
Post-primary tuberculous disease with bronchogenic spread in a 15-year-old girl. **a** Posteroanterior chest radiograph of shows ill-defined infiltrates in the left upper lobe (*encircled*). **b** Coronal reconstruction CT image demonstrates multiple centrilobular nodules (*arrows*) with linear branching opacities (tree-in-bud sign)

In 20% of post-primary tuberculosis cases, bronchogenic spread appears on radiographs as multiple, ill-defined micronodules in a segmental or lobar distribution, typically in the lower-lung zones [48]. High-resolution CT, the modality of choice, demonstrates centrilobular nodules ranging 2–4 mm with linear branching opacities (“tree-in-bud” sign) which represent caseous necrosis at and around terminal and respiratory bronchioles [49] (Fig. 20). Complete destruction of the entire lung or a large part of a lung is not uncommon in the end stage of tuberculosis. Secondary pyogenic or fungal infection can occur [47]. Miliary tuberculosis and tuberculomas might also be encountered in post-primary tuberculosis [2, 50].

## Approach to classification of intrathoracic tuberculosis

**Fig. 21:**
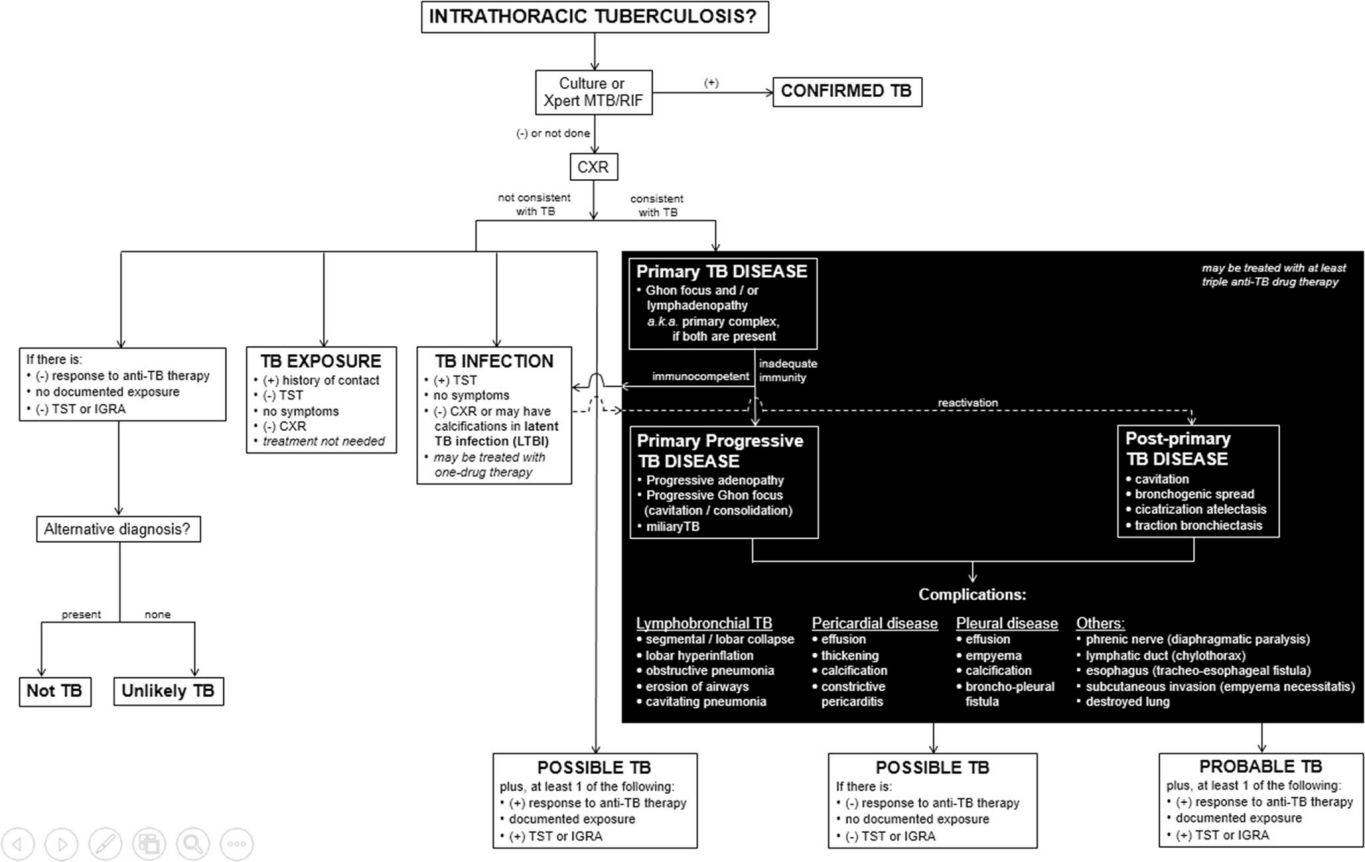
This is the proposed approach to classify intrathoracic tuberculosis in children. It includes clinical, laboratory and radiologic (*in black background*) components. The imaging characteristics follow the pathological processes and the possible complications that occur. The impression of the imaging findings should contain the main pathology (i.e. primary tuberculosis infection, primary progressive tuberculous disease or post-primary tuberculous disease) followed by the various complications present (e.g., primary progressive tuberculosis with lymphobronchial involvement and pleural disease). *CXR* chest radiograph*, IGRA* interferon-gamma release assay*, LTBI* latent tuberculous infection, *TB* tuberculosis/tuberculous, *TST* tuberculin skin test*, Xpert MTB/RIF* test to detect *Mycobacterium tuberculosis* and resistance to rifampicin [8]

We adapted the clinical and laboratory components of the classification from Graham et al. [3], Moyo et al. [4] and Triasih [5]. An imaging approach to interpretation is summarized in Fig. 21. The radiologic side of the algorithm is based on the pathological processes that occur in tuberculosis that result in various complications.

Whenever a child comes in for workup for a possible tuberculous infection or disease, it is important to be aware of the terminology and pathological pathways. The diagnosis or impression of the imaging findings should contain the main pathology (i.e. primary tuberculous infection, primary progressive tuberculous disease or post-primary tuberculous disease), followed by the various complications present (e.g., primary progressive tuberculosis with tracheobronchial involvement and pleural disease; Fig. 21).

## Pitfalls and limitations of imaging

Chest radiography is the primary screening tool in children suspected of having tuberculosis. It has, however, high intra- and inter-observer variability, with 74% specificity and 39% sensitivity [51] even in the best technical quality radiographs. A normal chest radiograph does not rule out tuberculosis [52]. CT offers excellent anatomical visualization [53], but because of its high cost and the higher radiation exposure, it is reserved for complicated cases [18].

Lymphadenopathy is the most common abnormality noted in children with primary tuberculosis [13], but it is not pathognomonic of tuberculosis because other infectious processes can present with lymphadenopathy. Large pulmonary vessels are sometimes erroneously identified as lymph nodes, leading to over-diagnosis. The inter-observer agreement is low (kappa −0.03 to 0.25) [5]. Knowledge and familiarization of the hilar anatomy are prerequisites prior to interpretation [52].

The abnormalities seen on chest radiographs resolve gradually and can worsen despite clinical improvement. Lymphadenopathy and parenchymal disease without or with calcifications can also persist for many months and even years even after proper treatment. Re-treatment might not be necessary, especially if the child is asymptomatic. Moreover, the calcifications do not equate with healed tuberculosis because these can indicate latency [52].

## Conclusion

Radiologic interpretation of pulmonary tuberculosis remains challenging. Classically, tuberculosis is classified as primary and post-primary tuberculosis, but the typical radiologic patterns are now complicated by overlapping imaging characteristics as well as occurrence of atypical features seen in immunocompromised children. It is important to differentiate infection and disease because treatments are different. The proposed standardized clinical and radiographic classification presented in this paper aims to provide helpful guides in the proper nomenclature of suspected tuberculosis patients. The pitfalls and limitations of imaging likewise caution both clinicians and radiologists to avoid erroneous interpretations and over-diagnosis of childhood tuberculosis.
